# A Randomized Prospective Study of Bowel Preparation for Colonoscopy with Low-Dose Sodium Phosphate Tablets versus Polyethylene Glycol Electrolyte Solution

**DOI:** 10.1155/2014/879749

**Published:** 2014-09-15

**Authors:** Erina Kumagai, Tomoyoshi Shibuya, Masae Makino, Takashi Murakami, Shiori Takashima, Hideaki Ritsuno, Hiroya Ueyama, Tomohiro Kodani, Hitoshi Sasaki, Kenshi Matsumoto, Naoto Sakamoto, Taro Osada, Akihito Nagahara, Tatsuo Ogihara, Sumio Watanabe

**Affiliations:** Department of Gastroenterology, Juntendo University School of Medicine, 2-1-1 Hongo, Bunkyo-ku, Tokyo 113-8421, Japan

## Abstract

Optimal bowel preparation is essential for the safety and outcome of colonoscopy. A solution containing polyethylene glycol (PEG) is often used as a bowel cleansing agent, but some patients are intolerant of PEG, and this may lead to discontinuation of colonoscopy. Sodium phosphates (NaP) tablets are designed to improve patient acceptance and compliance. The objective of this study was to compare bowel preparation efficiency and patient acceptance of a 30 NaP tablet preparation (L-NaP) and a 2 L PEG preparation. Patients were randomized into either the L-NaP or PEG group. The primary endpoint was the efficiency of colon cleansing as assessed by a validated four-point scale according to the Aronchick scale by endoscopists and was verified by blinded investigators. The secondary endpoints were patients' tolerability and acceptance. Colon-cleansing efficiency was not significantly different between the two preparations. However, patients' overall judgment was significantly in favor of L-NaP, reflecting better acceptance of L-NaP than PEG. Additionally, more patients favored L-NaP over PEG in a hypothetical future occasion requiring colonoscopy.

## 1. Introduction

Colorectal cancer (CRC) is one of the most common malignancies in the Western world. It is the second most frequent cause of mortality in Europe [1] and the USA [2, 3]. Recently, the incidence of CRC has been increasing in Japan; it is now the second in incidence rate and the third highest cause of mortality [4]. Colonoscopy is an established procedure for surveillance and evaluation of the gut status. Additionally, good bowel preparation is essential for observing and assessing the status of the colonic mucosa. Hitherto, an electrolyte solution containing polyethylene glycol (PEG) has been widely used as a cleansing agent for bowel preparation. However, a significant fraction of patients are intolerant of PEG solutions due to their unpleasant taste, which may lead to inadequate colon cleansing and even discontinuation of colonoscopy.

In light of the above background, a sodium phosphate (NaP) preparation has been developed to achieve better acceptance and improve outcomes of surveillance colonoscopy [5]. Several randomized trials and meta-analyses of PEG and NaP have indicated that NaP is safe, is well tolerated, is cost-effective, and is equally effective or better than PEG as a cleansing agent for bowel preparation [5–10]. However, conventional NaP tablets contain microcrystalline cellulose (MCC), which was found to be deposited in the colon, requiring additional irrigation. Therefore, MCC may interfere with the visualization of the mucosal lining during surveillance colonoscopy and has limited popularity among endoscopists. In 2011, an improved MCC-free NaP tablet became available in Japan with promising efficiency as a cleansing agent for bowel preparation.

We used Visiclear tablets for NaP (Zeria Pharmaceutical, Tokyo, Japan). In the USA, the NaP tablets used are Visicol tablets, which are produced by Salix Pharmaceutical. Visiclear and Visicol contain the same form of sodium phosphate at the same ratio. Further, each Visicol tablet contains 1.102 g sodium phosphate monobasic monohydrate and 0.398 g sodium phosphate dibasic anhydrous for a total of 1.5 g of sodium phosphate per tablet. The recommended dose of Visiclear tablets is 50 tablets (50 grams of sodium phosphate) taken orally with a total of 2 L of clear liquids. Patients take all 50 tablets on the day of the colonoscopy. NaP can be coadministered with a small dose of laxative when the dose of NaP has to be low. This prospective randomized pilot study represents the first endeavor to compare bowel preparation efficiency and patient acceptance of a 30 NaP tablet preparation with 2 L PEG solution plus mosapride and sennoside.

## 2. Materials and Methods

### 2.1. Patients and Setting

This was a prospective, randomized, investigator-blinded study with the aim of comparing a preparation containing 30 NaP tablets plus 15 mg of mosapride and sennoside (L-NaP) as a single dose with 2 L of PEG-electrolyte solution plus 15 mg of mosapride and sennoside as a single dose in patients requiring bowel preparation to undergo surveillance colonoscopy (see Table 1). The study was conducted at Juntendo University, Tokyo, from July 2011 to August 2013. Participating patients' age range was 20–74 years.

Patients were considered ineligible if they had any of the following conditions: being over 75 years of age, acute or chronic renal insufficiency (serum creatinine level >2.0 mg/dL), congestive heart failure, unstable angina, long QT syndrome, massive ascites, toxic megacolon, gastrointestinal obstruction, intestinal perforation, dysphasia, or a history of colorectal surgery. Also ineligible were patients who were taking medication known to influence renal function including diuretics, angiotensin-converting enzyme (ACE) inhibiters, angiotensin II receptor blockers (ARBs), nonsteroidal anti-inflammatory drugs (NSAIDs) or those who were judged as ineligible by the investigating physician.

### 2.2. Sample Size Calculation

The sample size calculation was based on the noninferiority of the L-NaP, assuming *α* = 0.05, *β* = 0.2, and *δ* = 0.1. A power analysis showed that if the outcome did not differ between the L-NaP tablet group and the PEG group, a sample of 42 patients in each group would be adequate to show no difference between the groups. Accordingly, a sample size of a total of 50 patients was required. Considering the possibility that the outcomes may differ between the L-NaP tablet and the PEG groups, a sample size of a total of 100 patients (50 patients per group) was the goal of this study.

### 2.3. Randomization and Blinding

A total of 100 eligible patients were randomly assigned to receive one of the two bowel preparation reagents described above by using a computer-generated random-number generating method. Patients were randomized in block sizes of two (L-NaP group and PEG group).

### 2.4. Procedures

On the day before colonoscopy, patients in both groups took a 5 mg mosapride citrate hydrate tablet (Gasmotin) before each meal and 24 mg sennoside (Pursennid) at bedtime. There was no meal restriction on the day before colonoscopy. At 4 to 6 h prior to the colonoscopy, L-NaP or PEG was taken as follows. In the L-NaP group, each patient ingested 5 NaP tablets (Visiclear, Zeria Pharmaceutical, Tokyo, Japan) orally every 15 min with 200 mL of clear liquid (water or green tea; total 30 tablets and 1.2 L liquid). In the PEG group, each patient took PEG solution (NIFLEC, Ajinomoto Pharmaceuticals, Tokyo, Japan) orally at a rate of approximately 1 L/h up to a total of 2 L. We encouraged all patients to drink water if they experienced thirst.

### 2.5. Ratings for Colon Cleansing

The primary endpoint was overall colon cleansing. Colon cleansing was evaluated by 2 independent gastroenterologists who were blinded to the treatment allocations. One of the gastroenterologists performed the colonoscopy and another evaluated the endoscopic images. The quality of colon cleansing was rated by a modified version of the Aronchick scale [11] as follows: “excellent” (greater than 90% of the mucosa was clearly seen; mostly liquid stool with minimal suctioning needed for adequate visualization); “good” (greater than 90% of the mucosa was clearly seen; mostly liquid stool, but significant suctioning needed for adequate visualization); “fair” (greater than 90% of the mucosa was clearly seen; a mixture of liquid and semisolid stool could be suctioned or washed); and “poor” (less than 90% of the mucosa was seen together with a mixture of semisolid and solid stool that could not be suctioned or washed). These ratings were classified into “adequate” (excellent or good) and “inadequate” (fair or poor) for the purpose of analysis. The quality of bowel cleansing was assessed for each segment of the colon ((i) cecum-ascending colon, (ii) transverse colon, (iii) descending colon, (iv) sigmoid colon, and (v) rectum).

### 2.6. Evaluations of Patients' Acceptance and Tolerance

The secondary endpoints of the study were patients' acceptance and tolerance of the two bowel preparation reagents. This information was acquired via a standardized questionnaire provided to the patients on the day of colonoscopy. The questionnaire required “yes,” “no,” or ordinal scale answers. Questions and possible answers were as follows. (A) How did you feel about this bowel preparation reagent compared with a previous preparation (if any)? This preparation was “easier” or “harder,” “cannot compare because I do not remember,” “cannot evaluate because this is my first colonoscopy,” and “cannot evaluate because experience was the same” or “not much different”. (B) How easy or difficult was it to ingest the preparations? “Very easy,” “easy,” “tolerable,” “difficult,” or “very difficult” (one item). (C) How did you feel about the fluid volume? “Not too much,” “a little too much,” or “too much.” (D) Did you feel a change in your physical condition? “Yes” or “no.” What kind of side effects did you experience? “Nausea,” “vomiting,” “abdominal pain,” or “abdominal distension.” (E) In the future, if you needed a colonoscopy, would you ask your doctor for the same preparation reagent again? “Fervently hope for the same preparation,” “hope for the same preparation,” “hope for the other preparation,” or “fervently hope for the other preparation.”

### 2.7. Ethical Considerations

Prior to the initiation of the study, the investigation protocol was reviewed and approved by the Institutional Review Board at the Juntendo University School of Medicine (the study site). Further, all participating patients provided informed consent after being informed of the purpose of the study and the nature of the procedures involved. The study was conducted with strict adherence to the Helsinki Declaration with extra care to avoid undue suffering.

### 2.8. Statistics

To compare differences in patients' demographic variables, Student's *t*-test (for age, disease duration, and total examination time), *χ* ^2^ test (for gender), Fisher's exact test (for purpose), and Wilcoxon rank-sum test (for examination frequency) were applied. The primary outcome was the outcomes of comparison between the two groups based on the Wilcoxon rank-sum test. For secondary endpoints, the Wilcoxon rank-sum test or the *χ* ^2^ test was used. A *P* value less than 0.05 was considered statistically significant. All analyses were done by SPSS Statistics Version 22 (IBM, Armonk, NY, USA).

## 3. Results

### 3.1. Baseline Observations

**Figure 1:**
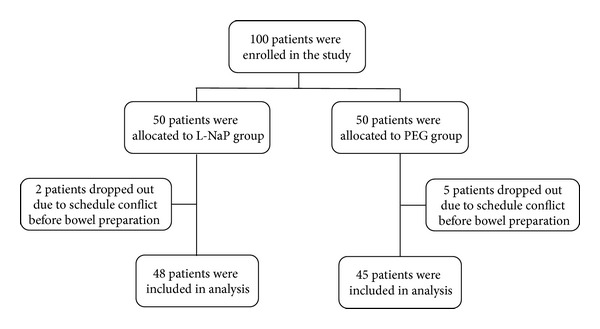
Flow diagram of patient recruitment and summary of treatment outcomes. A total of 100 patients were randomly assigned to two groups. Two patients in the sodium phosphate (NaP) tablet group and 5 patients in the polyethylene glycol solution (PEG) group did not undergo colonoscopy due to schedule conflicts before bowel preparation. They withdrew from the study. Therefore, 93 patients were included in the final analyses (48 in the L-NaP tablet group and 45 in the PEG group).

One hundred patients were included in this study and were randomly assigned to either the L-NaP (*n* = 50) or PEG group (*n* = 50) as seen in Figure 1. Two patients in the L-NaP group and 5 patients in the PEG group who had provided written informed consent could not undergo colonoscopy due to schedule complications before bowel preparation and withdrew from the study, leaving 93 patients available for the final analyses. There was no difference in age, gender, total examination time, indication for colonoscopy, or examination frequency between the two groups (Table 1).

### 3.2. Evaluation of Bowel Cleansing by Endoscopists

**Figure 2:**
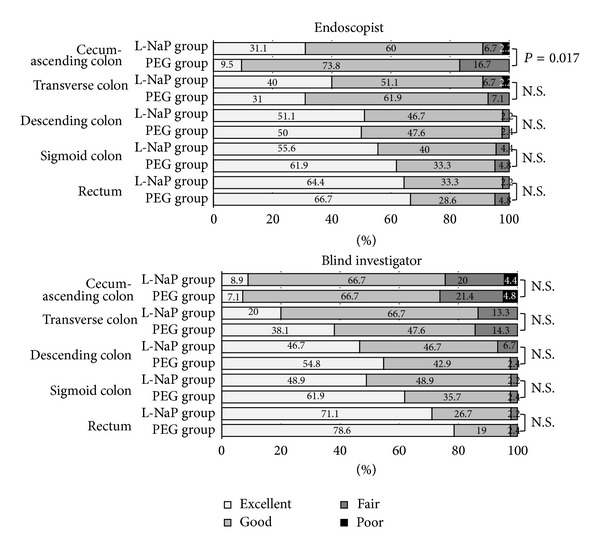
Efficiency of bowel cleansing in patients undergoing colonoscopy. Colon cleansing was evaluated by endoscopists and blinded investigators. As for the colon-cleansing effect in the cecum-ascending colon, L-NaP was rated to be superior to PEG. In other segments, there were no significant differences between the 2 reagents.

From the cecum to the ascending colon, adequate bowel preparation rates were 91.1% in the L-NaP group and 83.3% in the PEG group, with the cleansing score in the L-NaP group being significantly better than in the PEG group (*P* = 0.017, Figure 2). On the other hand, the cleansing rates for the transverse colon (L-NaP 91.1% versus PEG 92.9%), descending colon (97.8% versus 97.6), sigmoid colon (95.6% versus 95.2), and rectum (97.7% versus 95.3) did not differ significantly between the 2 groups (Figure 2).

### 3.3. Evaluation of Endoscopic Images

As stated above, endoscopic images were evaluated by a second independent endoscopist (blinded investigator) who was unaware of the treatment allocations. Adequate bowel preparation rates were not significantly different between the L-NaP and PEG groups in any segment of the colon (75.6% versus 73.8% in the ascending colon, 86.7% versus 85.7% in the transverse colon, 93.4% versus 97.7% in the descending colon, 97.8% versus 97.6% in the sigmoid colon, and 97.8% versus 97.6% in the rectum) as shown in Figure 2. The concordance rates between the endoscopist and blinded investigator were 80.2% in the ascending colon, 84.9% in the transverse colon, 95.4% in the descending colon, 97.7% in the sigmoid colon, and 96.6% in the rectum.

### 3.4. Patients' Acceptance and Tolerance of the Two Bowel Preparation Reagents

**Figure 3:**
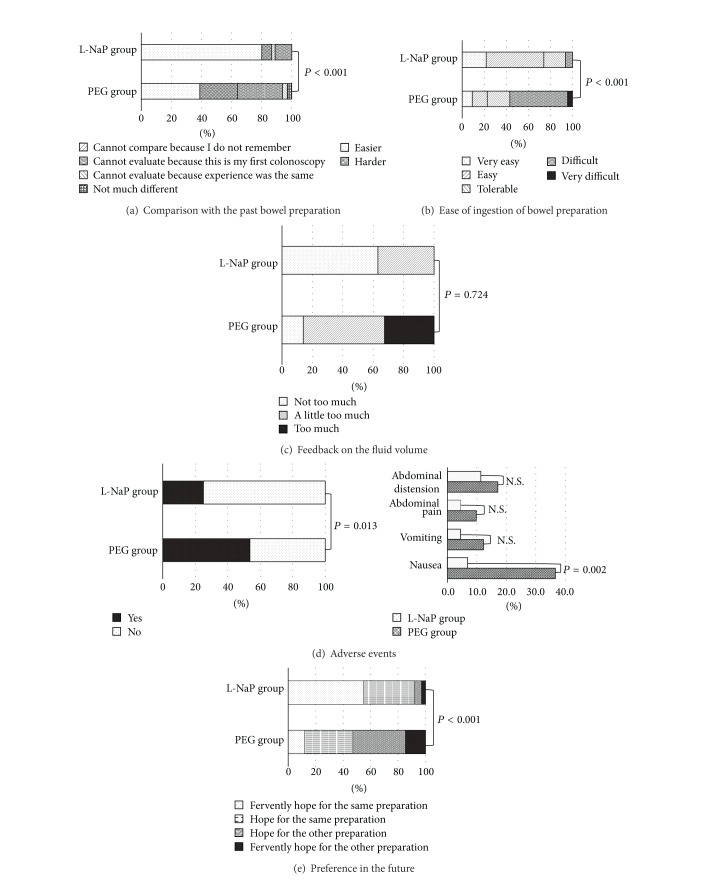
Outcomes of assessments of acceptability, tolerability, and safety.

As seen in Figure 3(a), 80% of patients in the L-NaP group reported that they had an easier bowel preparation experience than in the past in comparison with 38.9% of the patients in the PEG group (*P* < 0.001). Likewise, Figure 3(b) shows that, with regard to ease of ingestion of the bowel preparation, a greater fraction of patients in the L-NaP group rated the reagent as “very easy” or “easy” to ingest than patients in the PEG group: 73.9% versus 22.7% (*P* < 0.001). Further, Figure 3(c) shows that with regard to fluid volume, 32.6% of patients in the PEG group reported that the ingested volume was too much compared with 0% in the L-NaP group.

### 3.5. Safety

There was no adverse event requiring medical intervention in either group. The most common adverse events were “nausea,” “vomiting,” “abdominal pain,” and “abdominal distension” (Figure 3(d)). Among these, “nausea” was more common in the PEG group than in the L-NaP group (*P* = 0.002). Overall, a smaller fraction of patients in the L-NaP group experienced an adverse event than patients in the PEG group (*P* = 0.013).

### 3.6. Patient Preference

In the L-Nap group, 92.1% of patients expressed preference to receive the same preparation in the future (fervently hoped: 55.3% and hoped: 36.8%) compared with 47.1% of the patients in the PEG group (*P* < 0.001). Overall, the distributions of ratings of acceptability and tolerability for L-NaP were significantly better than for PEG regardless of the difference in fluid volume (Figure 3(e)).

## 4. Discussion

For gastroenterologists, colonoscopy has become an indispensable procedure for identifying and assessing disorders that commonly affect the large intestine. However, the outcomes of a colonoscopy procedure, whether it is just for undertaking surveillance or for the diagnosis and assessment of known conditions like ulcerative colitis, to a large extent depend on an adequate bowel preparation, which is a very tedious process. Additionally, clinical experience indicates that patients may respond unfavorably to the taste, to the odor, or to a large volume of cleansing agent to be ingested prior to colonoscopy. Potentially, this may lead to low patient compliance and acceptance of surveillance colonoscopy. Hitherto, PEG has often been used as an orally ingestible colon-cleansing solution in Japan and elsewhere. However, many patients are not able to complete the PEG dose due to its unpleasant taste, odor, and the large volume, often 2 L, needed for colon cleansing. Accordingly, NaP as an alternative bowel cleansing reagent was developed to overcome the limitations of PEG-based bowel cleansing. Preliminary evaluation trials [5–10, 12] reported a good safety profile and favorable patient response to NaP [13]. For polyp detection, the positive rate for L-NaP (40%) was very much higher than for PEG (19%) [12]. Most notably, the NaP preparation was associated with a detection rate of 38.6% for polyps with a diameter <5 mm compared with 18.8% for a PEG-based bowel cleansing reagent [14].

In the present study, we found that a low-dose NaP-based preparation had a bowel-cleansing action similar to a PEG-based preparation but with better tolerability and safety profile. As far as we are aware, this is the first study that has evaluated the clinical relevance of low-dose NaP tablets for bowel cleansing prior to colonoscopy. As mentioned above, one limitation of PEG-based bowel cleansing is the large volume of the reagent, usually 2 L, which patients need to ingest. In this regard, 1.2 L of liquid taken with NaP tablets represents a significant reduction versus 2 L of PEG solution. However, for Japanese patients who are relatively smaller than patients in Europe or the USA, the 50 NaP tablets with 2 L of liquid that were used in earlier trials can be excessive. Generally speaking, the large volume of fluid to be ingested contributes to lower tolerability and compliance. Accordingly, we thought that there was a need to reduce the fluid volume to improve acceptability and tolerability without compromising the cleansing effect. Additionally, the original prototype NaP tablets contained MCC, which is known to impair visualization of the mucosal lining during colonoscopy [15]. The MCC-free tablet formulation used in this study was expected to allow better cleansing scores compared with the MCC-containing tablet formulation [16]. Both the lower liquid volume and the MCC-free NaP formulation should be favorable features in clinical settings.

Coadministration of laxatives, including sennoside and bisacodyl, with lavage solution has been reported to improve colon cleansing in advance of colonoscopy. Addition of these adjunctive agents also has allowed a reduction in the PEG solution volume without decreasing cleansing efficiency. Alternatively, coadministration of mosapride with PEG produced better acceptability and tolerability [17]. Likewise, mosapride citrate has been reported to be a promising safe adjunct to PEG-electrolyte solution and has been associated with an improved quality of bowel preparation [18]. In Japan, 2 L of PEG is the standard preparation volume prior to colonoscopy. Originally, coadministration of a mild laxative with 2 L of PEG has been used for colon preparation. Based on this background, we studied the cleansing effect of L-NaP with mosapride and sennoside focusing on patients' acceptability and tolerability. In this study, with respect to the outcome of the endoscopist's evaluation, the L-NaP group had a significantly better result than the PEG group from the cecum to the ascending colon. In other segments, the L-NaP preparation was similar to 2 L PEG-based preparation. In the blinded investigator's evaluation, the outcome with L-NaP was equivalent to that of the 2 L PEG preparation. The concordance rate of the evaluation between the endoscopist and the blinded investigator was high for the distal colon, while in the ascending colon and the transverse colon, the concordance rates were low. The reason may be that fecal residue became buried in the proximal colon which has deep haustration, potentially compromising photo precision. In all situations examined, the performance of the L-NaP preparation was not inferior to that of the 2 L PEG preparation.

However, the L-NaP preparation was superior to PEG with respect to patients' tolerability and acceptability. In the L-Nap group, 80% of the patients answered that it was easier than past bowel preparations, and 92.1% of patients wished to receive the same preparation for a future endoscopy. Also, in this study, there was no adverse event requiring treatment in either group. The most common adverse event was “nausea,” which was more frequent in the PEG group than in the L-NaP group. Accordingly, the overall rate of adverse events in the L-NaP group was lower than in the PEG group. We considered that the cause of the adverse events in both groups was related to the intake of a large volume of cleansing fluid in the preparation.

In the present study, we excluded patients with preexisting renal dysfunction based on several reports regarding the side effects of NaP, including hypernatremia [19, 20], hypocalcemia [21], hyperphosphatemia [22–27], and acute phosphate nephropathy in elderly patients [28]. Sodium phosphate is contraindicated in patients with preexisting renal disease [29] and, therefore, renal function should be assessed before and after colonoscopy in patients who receive NaP. The bottom line is that NaP should not be given to patients on medications that can affect renal function such as diuretics, ACE inhibitors, ARBs, and NSAIDs.

A possible limitation of this study is that in both groups there were only a few patients with severe constipation. It is possible that, for patients with severe constipation, a bowel cleansing solution with low-dose NaP may be inadequate. We believe that, from the start, patients with severe constipation should take a full dose of bowel preparation. The second limitation of this study is that it was a single-center trial with a small number of patients. Further larger studies are warranted to fully evaluate the colon cleansing effect and acceptability of L-NaP tablets. Finally, we did not evaluate laboratory abnormalities. However, both mosapride and sennoside were taken at regular doses, which have known safety profiles.

In conclusion, the low-dose NaP tablet preparation had similar bowel cleansing efficiency to the PEG-based preparation but had better safety and tolerability profiles, and this was reflected in good patient compliance and preference. However, limitations may apply in using an L-NaP-based preparation in patients with a renal disorder. With these restrictions in mind, we believe that L-NaP is a clinically relevant bowel preparation reagent in patients without underlying kidney or heart insufficiency.

## Figures and Tables

**Table 1:** Patients' demographic features and indications for surveillance colonoscopy.

	NaP group	PEG group	*P* value
Number	48	45	
Mean age (range)	45.2 ± 13.0 (21–69)	46.2 ± 13.6 (25–71)	NS *t*-test
Men : women	27 : 21	26 : 19	NS *χ* ^2^-test
Intubation time (range)	10.1 ± 6.3 (2–30)	10.0 ± 5.0 (2–20)	NS *t*-test
Total examination time (range)	20.3 ± 6.8 (8–39)	17.7 ± 5.9 (10–30)	NS *t*-test
Purpose			
Cancer surveillance or screening	17	17	NS Fisher's exact test
Positive fecal occult blood test or rectal bleeding	5	4	
Inflammatory bowel disease	20	13	
Changes in bowel habit or pain	6	8	
Examination frequency			
First time	7	10	NS Wilcoxon rank-sum test
Second time	9	8	
Third time	5	13	
Over four times	25	13	
